# Disrupted sphingolipid metabolism following acute clozapine and olanzapine administration

**DOI:** 10.1186/s12929-018-0437-1

**Published:** 2018-05-02

**Authors:** Katrina Weston-Green, Ilijana Babic, Michael de Santis, Bo Pan, Magdalene K. Montgomery, Todd Mitchell, Xu-Feng Huang, Jessica Nealon

**Affiliations:** 1 Centre for Medical and Molecular Biosciences, and School of Medicine, Faculty of Science, Medicine and Health, University of Wollongong, Wollongong, NSW 2522 Australia; 2 Illawarra Health and Medical Research Institute, Wollongong, NSW 2522 Australia; 3 Illawarra and Shoalhaven Local Health District, Wollongong, NSW 2500 Australia; 4 Department of Pharmacy, Yangzhou University Medical Academy, Yangzhou, 225001 Jiangsu China; 5 Department of Physiology, School of Biomedical Sciences, Monash University, Clayton, VIC 3800 Australia; 6 Mass Spectrometry User Resource and Research Facility, University of Wollongong, Wollongong, NSW 2522 Australia

**Keywords:** Antipsychotic, Sphingolipid, Ceramide, Sphingomyelin, ER stress, Hyperglycemia

## Abstract

**Background:**

Second generation antipsychotics (SGAs) induce glucometabolic side-effects, such as hyperglycemia and insulin resistance, which pose a therapeutic challenge for mental illness. Sphingolipids play a role in glycaemic balance and insulin resistance. Endoplasmic reticulum (ER) stress contributes to impaired insulin signalling and whole-body glucose intolerance. Diabetogenic SGA effects on ER stress and sphingolipids, such as ceramide and sphingomyelin, in peripheral metabolic tissues are unknown. This study aimed to investigate the acute effects of clozapine and olanzapine on ceramide and sphingomyelin levels, and protein expression of key enzymes involved in lipid and glucose metabolism, in the liver and skeletal muscle.

**Methods:**

Female rats were administered olanzapine (1 mg/kg), clozapine (12 mg/kg), or vehicle (control) and euthanized 1-h later. Ceramide and sphingomyelin levels were examined using electrospray ionization (ESI) mass spectrometry. Expression of lipid enzymes (ceramide synthase 2 (CerS2), elongation of very long-chain fatty acid 1 (ELOVL1), fatty acid synthase (FAS) and acetyl CoA carboxylase 1 (ACC1)), ER stress markers (inositol-requiring enzyme 1 (IRE1) and eukaryotic initiation factor (eIF2α) were also examined.

**Results:**

Clozapine caused robust reductions in hepatic ceramide and sphingolipid levels (*p* < 0.0001), upregulated CerS2 (*p* < 0.05) and ELOVL1 (+ 37%) and induced significant hyperglycemia (vs controls). In contrast, olanzapine increased hepatic sphingomyelin levels (*p* < 0.05 vs controls). SGAs did not alter sphingolipid levels in the muscle. Clozapine increased (+ 52.5%) hepatic eIF2α phosphorylation, demonstrating evidence of activation of the PERK/eIF2α ER stress axis. Hepatic IRE1, FAS and ACC1 were unaltered.

**Conclusions:**

This study provides the first evidence that diabetogenic SGAs disrupt hepatic sphingolipid homeostasis within 1-h of administration. Sphingolipids may be key candidates in the mechanisms underlying the diabetes side-effects of SGAs; however, further research is required.

## Background

Second generation antipsychotics (SGAs), such as olanzapine and clozapine, are approved to treat schizophrenia and bipolar disorder. SGAs are also increasingly prescribed for a growing number of illnesses in adults and children [1]. Unfortunately, olanzapine and clozapine can cause serious metabolic side-effects, including weight gain, obesity, and prolonged hyperglycemia that can lead to insulin resistance and diabetes mellitus (DM) [2]. Glucometabolic side-effects can occur independently of weight gain, suggesting that SGAs could disrupt glucose metabolism directly. Despite almost two decades of scientific research [3], the precise mechanisms underlying the hyperglycaemic side-effects of these drugs remain unclear. In addition, reports suggest a recent drying of the antipsychotic drug development pipeline [4]. Therefore, the hyperglycemia side-effects of SGAs continue to pose a critical therapeutic challenge for mental illness and further mechanistic research is required.

Peripheral tissues, such as the liver, skeletal muscle and adipose tissues, play important roles in metabolic homeostasis and a growing body of literature demonstrates that SGAs target these tissues to induce metabolic side-effects [5–7]. Clinical and experimental studies have shown that inappropriate hepatic glucose production and peripheral insulin resistance are major contributors to olanzapine- and clozapine-induced prolonged hyperglycemia. For example, evidence demonstrates that olanzapine and clozapine induce an imbalance in glucagon and insulin [8–10], favoring hepatic gluconeogenesis, glycogenolysis and suppression of glycogen synthesis. Other studies show that clozapine induces insulin resistance by directly impairing insulin receptor (IR) activity and downstream signalling events (ie reduced IR substrate-1 (IRS-1) phosphorylation) in mouse skeletal muscle [11], while olanzapine was shown to upregulate mRNA expression of key gluconeogenic enzymes, leading to hyperglycemia and insulin resistance in rats [12]. Olanzapine and clozapine also induce the development of hepatic steatosis, with reports of a reduction in hepatic lipolytic capacity (such as reduced hormone-sensitive lipase (HSL)), reduced fatty acid oxidation, as well as increases in lipogenesis (increased gene expression of fatty acid synthase (FAS) and acetyl-CoA carboxylase (ACC), with lipogenic gene expression commonly driven by a SGA-induced upregulation of sterol regulatory element-binding protein (SREBP)) [5, 13]. In addition, hepatic lipogenesis and subsequent lipid accumulation can also be driven by activation of endoplasmic reticulum (ER) stress, thereby also contributing to glucometabolic imbalance and leading to reduced insulin signalling and whole-body glucose intolerance [7, 14, 15]. ER stress activates unfolded protein response (UPR) pathways, which act to reduce the demand for protein folding in an effort to reduce cellular stress and avert cell apoptosis [16]. Stress in the ER lumen activates three transmembrane proteins: inositol-requiring enzyme 1 (IRE1), protein kinase RNA-like endoplasmic reticulum kinase (PERK) and activating transcription factor 6 (ATF6) that signal to monitor ER stress. PERK transiently inhibits eukaryotic initiation factor (EIF-2α) through phosphorylation, while IRE1 triggers other cell fate signalling pathways, such as c-Jun N-terminal kinase (JNK) that is also a central mediator of insulin signalling and implicated in insulin resistance [7, 14]. In addition, a major downstream intermediate of the IRE1 pathway, X-box binding protein 1, upregulates hepatic lipogenesis by acting as a transcription factor for lipogenic genes [17]. However, the effect of diabetogenic SGAs on these enzymes is unknown.

Sphingolipids also play an important role in glycaemic balance and insulin resistance (see reviews by [18, 19]). Ceramides are a sphingolipid sub-class that contain a fatty acyl chain attached to a sphingosine backbone via an amide linkage [20, 21]. Ceramide is synthesised through several metabolic pathways, including the de novo pathway that produces ceramide in the ER and the salvage pathway where degradation of complex sphingolipids eventually produces ceramide. Ceramide synthesis via the de novo pathway involves ceramide synthase enzymes (CerS1–6) that produce different ceramide species based on their fatty acyl chain length [22]. The CerS2 isoform yields carbon (C) 22–24 ceramide species and C24 ceramides are reportedly one of the most abundant species in the body [22–24]. CerS2 also regulates the elongase enzyme, elongation of very long-chain fatty acid 1 (ELOVL1), which synthesises C18-C26 fatty acid chains using C16 as a substrate [25]. Ceramide production can be inhibited by fatty acid synthase (FAS), a key enzyme in the synthesis of the lipid precursor palmitate [4]. Following production in the ER, ceramide can be converted into sphingomyelin in the Golgi [22]. Ceramide and sphingomyelin are essential structural components of the plasma cell membrane and are bioactive players in numerous cell processes, including apoptosis and regulation of protein phosphorylation by ceramide in-vitro [22]. Complex sphingolipids are required for ER homeostasis and a reduction in sphingolipid levels has been shown to initiate cell death via ER stress-mediated pathways [26]. Ceramide inhibits glycogen synthesis and insulin-stimulated glucose uptake, blockade of ceramide synthesis improves insulin sensitivity and glucose homeostasis, and levels of ceramide species are higher in skeletal muscle of obese insulin-resistant subjects compared to lean controls [27]. Interestingly, sphingolipids are also altered in schizophrenia, for example increased ceramide levels have been reported in the white matter of the pre-frontal cortex [28] (also see review by Castillo et al. [29]), and in response to chronic haloperidol treatment, a first generation antipsychotic drug [30]. Altered sphingolipids in key glucometabolic tissues, such as the liver and skeletal muscle, may contribute to the disruption in glucose homeostasis that leads to hyperglycemia and insulin-resistance side-effects; however, this has not previously been examined. The aim of this study was to investigate the acute effects of olanzapine and clozapine administration on the levels of ceramide and sphingomyelin in the liver and skeletal muscle, as well as protein expression of key enzymes involved in lipid and glucose metabolism.

## Methods

### Ethics

This study was approved by the Animal Ethics Committee of the University of Wollongong (AE13/19). Procedures complied with the Australian Code of Practice for the Care and Use of Animals for Scientific Purposes [31]. Efforts were made to minimise animal number and reduce suffering throughout the experimental process.

### Animals and treatment

Adult female Sprague Dawley rats (~ 200-225 g, 9 weeks) were obtained from the Animal Resource Centre (Perth, WA, Australia) and pair-housed at 22 °C (12 h dark-light cycle, photophase 07.00 h) with ad libitum access to water and standard laboratory chow (3.9 kcal/g; fat 10%, carbohydrates 74% and protein 16%) throughout the study. Female rats were selected based on the increased sensitivity of women to antipsychotic-induced metabolic side-effects in the clinical scenario [32], a phenomenon also apparent in female rats [33]. Rats were habituated for one-week to facilitate environmental acclimatisation. Following an overnight fast (13 ± 2 h), rats were administered an acute dose of olanzapine (Zyprexa, Lilly, Indianapolis, USA) (1 mg/kg), clozapine (Clozaril, Novatis, Basel, Switzerland) (12 mg/kg), or vehicle (control) (*n* = 12/group), as we have previously reported [34–39], then euthanized one-hour post-treatment via CO_2_ asphyxiation. Dosages were selected based on human clinical doses, translated to animal equivalent doses by considering differences in body surface area between species [40], and our previous reports [34–39]. The 1-h time period was used in order to determine the immediate (acute) drug effects on the parameters examined. Blood samples were immediately obtained and centrifuged (1000 g, 4 °C, 10 mins), then plasma was analysed in duplicate for glucose concentrations using commercial colorimetric glucose oxidase assay kits (Cayman Chemical, MI, USA). Post-mortem liver and skeletal muscle (gastrocnemius) tissues were dissected and frozen in liquid nitrogen, then stored at − 80 °C for analysis.

### Sphingolipid extraction and analysis by mass spectrometry

The lipid extraction and mass spectrometry methods were based on a previous study from our laboratory [41]. Briefly, lipids were extracted using a biphasic methyl-*tert*-butyl ether (MTBE) / methanol (HPLC grade, Thermo Scientific, Scoresby, VIC Australia) lipid extraction technique, whereby 5 mg of tissue was homogenised (*n* = 6–7 / group) in 300 μL methanol and butylated hydroxytoluene (MeOH+ 0.01% BHT) containing internal standards (SM (d18:0/12:0) 25 μM and Cer (d18:1/17:0) 5 μM, Avanti Polar Lipids, AL, USA; 10 μL/mg tissue) for quantification, using a ceramic bead homogeniser (Fast Prep-24, MP Biomedical, NSW, Australia; 6 m/s for 40 s). Residual tissue was removed from the beads using an additional 100 μL MeOH+ 0.01% BHT, then 920 μl of MTBE was added to the homogenate. Tubes were rotated for one hour at 4 °C, then 230 μL of 150 mM ammonium acetate (NH_4_OAc) was added and samples were briefly vortexed then centrifuged (2000 g, 5 min). The upper organic phase containing the lipids was collected into a glass vial without disturbing the lower aqueous phase. The extracted samples underwent further base hydrolysis to remove highly abundant phospholipids and allow detection of less abundant ceramide and sphingomyelin species. A 200 μL aliquot of extracted sample was combined with MeOH+ 0.01% BHT and 0.7 M sodium hydroxide. Samples were rotated (2 h, room temperature) then re-extracted using the same MTBE and NH_4_OAc methods above. Samples were vortexed then centrifuged (2000 g, 5 min). The upper organic phase containing the extracted lipid molecules of interest was removed and diluted 50-fold in MeOH and chloroform (CHCl_3_) (2:1 vol/vol) with 5 mM NH_4_OAc. Diluted extract (30 μL) was pipetted into a 96-well plate and heat-sealed with foil to prevent evaporation of the solvent. The lipid extracts were analysed by electrospray ionization (ESI) mass spectrometry using a hybrid triple quadrupole ion trap mass spectrometer (QTRAP® 5500 AB SCIEX, Framingham Massachusetts, USA) equipped with an automated chip-based nano-electrpospray source (Triversa Nanomate®, Advion, Ithaca, NY, USA), as previously described [41, 42]. Lipid extracts were analysed using precursor ion scans in positive ion mode ([M + H]^+^) using a mass to charge ratio (*m/z*) of 264.4 with a collision energy of 35 for ceramide, and a *m/z* of 184.1 and collision energy of 40 for sphingomyelin, respectively. Further ESI settings specific to the QTRAP® 5500 were used as previously reported [41]. Sphingolipid species were quantified against the internal standards using AB SCIEX Lipidview™ Software (Framingham, Massachusetts, USA) with the following processing settings, a mass tolerance of 0.5 Da, a minimum intensity of 0.1% and a minimum signal-to-noise ratio of 25, as we have previously described [41, 43].

### Western blot

Levels of protein expression in the tissue samples (*n* = 4–6 / group) were examined using standard western blot techniques, as previously described [44, 45]. Briefly, whole tissue samples were homogenized (Precellys, Sapphire Bioscience Australia) at 6 m/sec for 30 s in RIPA buffer (65 mmol/l Tris, 150 mmol/l NaCl, 5 mmol/l EDTA, pH 7.4, 1% (*v*/v) NP-40 detergent, 0.5% (*w*/*v*) sodium deoxycholate, 0.1% (w/v) SDS, 10% (v/v) glycerol, containing 25 μg/ml leupeptin, 10 μg/ml aprotinin, 2 mmol/l sodium orthovanadate, 10 mmol/l NaF and 1 mmol/l polymethylsulphonyl fluoride) [44]. Proteins were resolved using SDS-PAGE techniques and immunoblotting was performed using the following antibodies: total ACC (cat 3662), phosphorylated ACC (cat 3661), total Elf2a (cat 9722) and phosphorylated Elf2a (cat 3398) FAS (cat 3180) and total IRE1 (cat 3294) (Cell Signaling Technology, MA, USA), phosphorylated IRE1 (cat ab48187) (Abcam, Melbourne, Australia), while CerS2 (cat ARP32609) and Elovl1 (cat ARP49759) antibodies were purchased from Aviva Systems Biology (San Diego, CA, USA) Bands of interest were quantified by densitometry using ImageJ 1.44p software (NIH, USA).

### Glycogen content

Liver glycogen content was assayed using a phenol-sulphuric acid calorimetric method previously described [46]. Briefly, 50 mg of liver was digested in 200 μL of 1 M KOH at 70 °C, followed by addition of 75 μL of saturated Na_2_SO_4_ and 1.725 mL of 95% ethanol to precipitate glycogen. Samples were centrifuged at 13000 rpm for 10 min at 4 °C, and the glycogen pellet resuspended in 200 μL of H_2_O. Once resuspended, 1.8 mL of 95% ethanol was added, and the samples centrifuged again. The glycogen pellet was air dried, resuspended in 500 μL of a solution containing 0.3 mg/mL amyloglucosidase (Sigma-Aldrich, Castle Hill, NSW Australia) in 0.25 M acetate buffer (pH 4.75), and the glycogen digested into glucosyl unit overnight at 37 °C. Glucose was measured using a commercial glucose oxidase kit (Sigma-Aldrich).

### Statistical analysis

Statistical analysis was performed using SPSS Statistics software (version 21, IBM, IL, USA). Data was tested for normality using Shapiro-Wilk W tests. Outliers of ±2 SD were removed. Analysis of Variance (ANOVA) tests were employed to examine treatment effects on levels of plasma glucose, total sphingolipids and protein expression. Multivariate ANOVA (MANOVA) tests were used to examine treatment effects on levels of ceramide and sphingolipid species. Post-hoc analysis was performed using Bonferroni tests for multiple comparisons. Significance was accepted if *p* < 0.05.

## Results

### Fasting plasma glucose levels

**Fig. 1:**
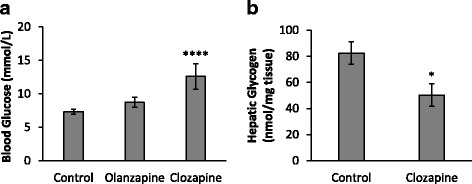
Glucose and Glycogen: (**a**) Fasting blood glucose levels (mmol/L) and (**b**) hepatic glycogen content (nmol/mg tissue) in female Sprague Dawley rats following acute administration of olanzapine (1 mg/kg), clozapine (12 mg/kg) or vehicle (control). Data presented as mean ± SEM, (*n* = 11–12/group). *p < 0.05 vs controls, ****p < 0.0001 vs controls

An ANOVA revealed a significant effect of treatment on fasting plasma glucose levels (F_2,35_ = 15.06, *p* < 0.0001), with the clozapine group exhibiting significant hyperglycemia compared to the controls (*p* < 0.0001); however, no significant differences in glucose levels were observed in the olanzapine treatment group (*p* > 0.05 vs controls) (Fig. 1). In order to investigate a potential mechanism underlying the clozapine-induced hyperglycaemia, we examined liver glycogen levels and observed a decrease (− 39%) in glycogen content in the clozapine group compared to the controls (*p* < 0.05) (Fig. 1).

### Sphingolipid levels

#### Ceramide levels

**Fig. 2:**
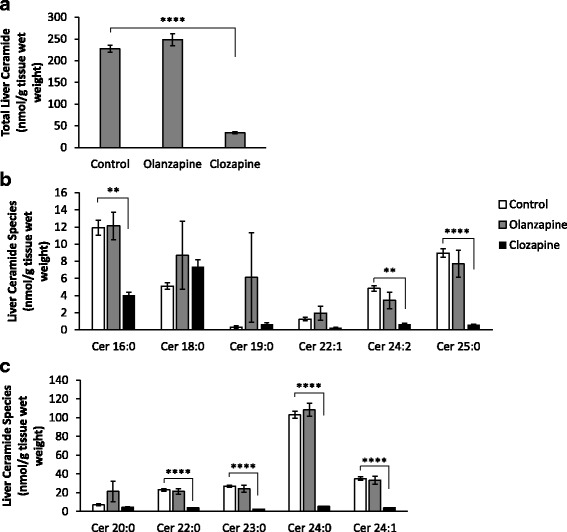
Hepatic Ceramide: Levels of (**a**) total ceramide, (**b**) lower abundance ceramide species and (**c**) higher abundance ceramide species (nmol/g tissue wet weight) in the rat liver following acute administration of olanzapine (1 mg/kg), clozapine (12 mg/kg) or vehicle (control). Data presented as mean ± SEM, (*n* = 6–7/group). **p < 0.01 vs controls, ****p < 0.0001 vs controls

There was a significant effect of treatment on total ceramide concentration in the liver (F_2,16_ = 131.59, *p* < 0.0001), with reduced levels following clozapine treatment compared to the controls (*p* < 0.0001). In contrast, no difference in ceramide concentration was observed in the olanzapine group (*p* > 0.05 vs controls) (Fig. 2). Further examination of individual ceramide species identified a lower level of Cer 16:0, Cer 24:2 (*p* < 0.01), Cer 22:0, Cer 23:0, Cer 24:0, Cer 24:1, Cer 25:0 (*p* < 0.0001) in the clozapine group compared to the controls (Fig. 2). There were no differences in the levels of Cer 18:0, Cer 19:0, Cer 20:0, Cer 22:1 in the clozapine group or in any of the ceramide species in the olanzapine group compared to the controls (all *p* > 0.05) (Fig. 2). We also examined treatment effects in the skeletal (gastrocnemius) muscle and found no significant effect of treatment on total ceramide (F_2,16_ = 4.09, *p* < 0.05) or individual ceramide species in the skeletal muscle (*p* > 0.05) (Table 1).

**Table 1:** Ceramide and Sphingomyelin in Skeletal Muscle: Levels of ceramide and sphingomyelin (nmol/g tissue wet weight) in the rat skeletal muscle (gastrocnemius) following acute administration of olanzapine (1 mg/kg), clozapine (12 mg/kg) or vehicle (control). Data presented as mean ± SEM, (*n* = 6–7/group)

	Control			Olanzapine			Clozapine		
Ceramide									
Total	49.08	±	1.19	33.96	±	6.03	47.65	±	2.81
Cer 16:0	3.96	±	0.72	2.05	±	0.38	3.77	±	1.02
Cer 18:0	22.71	±	1.84	18.75	±	2.50	24.77	±	0.91
Cer 19:0	0.79	±	0.03	0.60	±	0.17	0.82	±	0.07
Cer 20:0	3.40	±	0.11	2.93	±	0.27	2.33	±	0.33
Cer 22:0	3.35	±	0.40	3.28	±	0.32	2.65	±	0.28
Cer 22:1	0.31	±	0.15	0.20	±	0.10	0.37	±	0.14
Cer 23:0	2.17	±	0.29	2.14	±	0.03	1.66	±	0.23
Cer 24:0	7.08	±	0.97	6.71	±	0.68	6.08	±	0.93
Cer 24:1	5.32	±	0.40	3.46	±	0.23	4.49	±	0.70
Cer 24:2	0.84	±	0.10	0.44	±	0.21	0.71	±	0.17
Cer 25:0	0.30	±	0.14	0.31	±	0.14	0.30	±	0.15
Sphingomyelin									
Total	521.23	±	20.74	409.07	±	69.83	472.09	±	17.39
SM 16:0	59.07	±	12.48	51.95	±	9.07	64.12	±	8.68
SM 17:0	5.98	±	0.45	5.35	±	0.23	5.91	±	0.48
SM 18:0	241.68	±	10.63	213.19	±	20.40	253.40	±	9.12
SM 18:1	11.29	±	0.59	9.44	±	1.29	10.44	±	0.39
SM 19:1	4.78	±	0.18	4.57	±	0.22	4.78	±	0.18
SM 20:0	25.45	±	3.31	23.43	±	2.63	15.27	±	1.61
SM 22:0	37.96	±	5.27	43.44	±	6.70	23.39	±	2.75
SM 22:1	4.51	±	0.98	4.68	±	1.09	2.68	±	0.88
SM 23:0	14.60	±	1.48	18.36	±	2.68	10.25	±	1.09
SM 24:0	42.86	±	4.73	48.30	±	7.24	29.11	±	2.39
SM 24:1	66.81	±	8.28	49.84	±	2.33	45.62	±	4.91
SM 24:2	8.69	±	0.58	7.06	±	0.25	7.12	±	1.02

#### Sphingomyelin levels

**Fig. 3:**
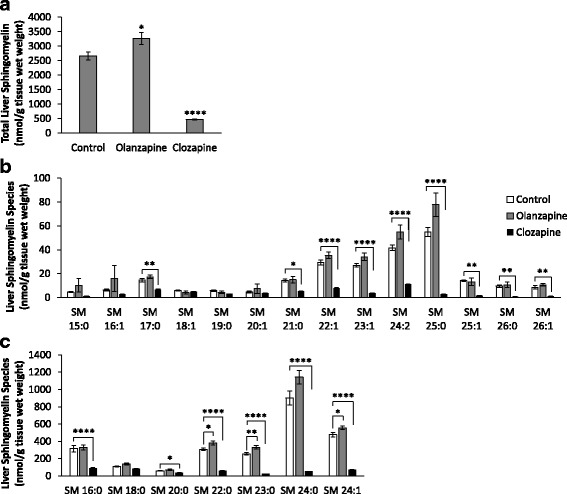
Hepatic Sphingomyelin: Levels of (**a**) total sphingomyelin, (**b**) lower abundance sphingomyelin species and (**c**) higher abundance sphingomyelin species (nmol/g tissue wet weight) in the rat liver following acute administration of olanzapine (1 mg/kg), clozapine (12 mg/kg) or vehicle (control). Data presented as mean ± SEM, (*n* = 6–7/group). *p < 0.05 vs controls, **p < 0.01 vs controls, ****p < 0.0001 vs controls

A significant treatment effect on total sphingomyelin levels was identified (F_2,16_ = 90.43, *p* < 0.0001), with lower levels in the clozapine group (*p* < 0.0001) and higher total sphingomyelin levels in the olanzapine group (*p* < 0.05) compared to the controls (Fig. 3). Analysis of sphingomyelin species revealed a significant decrease in the levels of SM 20:0, SM 21:0 (*p* < 0.05), SM 17:0, SM 25:1, SM 26:0, SM 26:1 (*p* < 0.01), SM 16:0, SM 22:0, SM 22:1, SM 23:0, SM 23:1, SM 24:0, SM 24:1, SM 24:2 and SM 25:0 (*p* < 0.0001) in the clozapine group compared to the controls (Fig. 3). There was no treatment effect on the levels of SM 15:0, SM 16:1, SM 18:0, SM 18:1, SM 19:0 or SM 20:1 (all *p* > 0.05) (Fig. 3). On the other hand, olanzapine significantly increased levels of SM 22:0, SM 24:1 (*p* < 0.05) and SM 23:0 (*p* < 0.01), while the remaining sphingomyelin species were unchanged by olanzapine treatment (Fig. 3). Similar to ceramide analysis, we then investigated whether changes in sphingomyelin levels were apparent in the skeletal (gastrocnemius) muscle and found no treatment effects on total sphingomyelin (F_2,16_ = 4.09, *p* < 0.05) or sphingomyelin species (*p* > 0.05) (Table 1).

### Protein expression of metabolic markers in the liver

#### Lipid metabolism

**Fig. 4:**
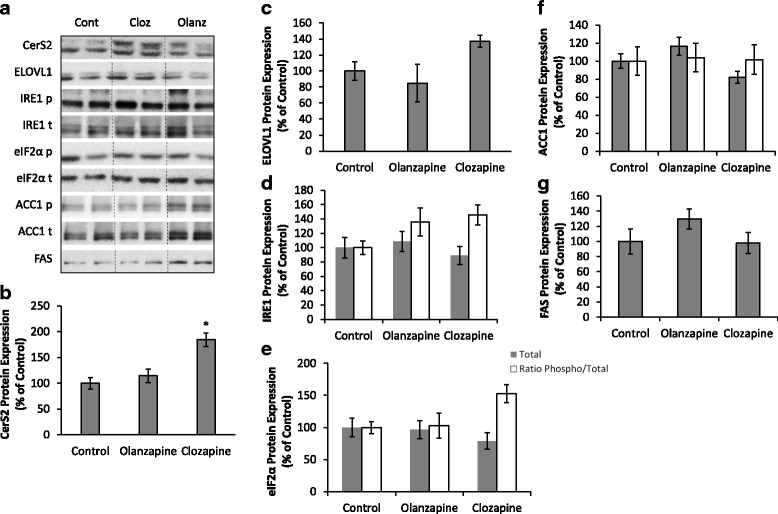
Western Blot: Examples of Western blot protein expression are shown in (**a**). Levels of (**b**) ceramide synthase 2 (CerS2), (**c**) elongation of very long chain fatty acids 1 (ELOVL1), (**d**) inositol-requiring enzyme (IRE1) and (**e**) eukaryotic initiation factor 2 (eIF2α), (**f**) acetyl CoA carboxylase (ACC1) and (**g**) fatty acid synthase (FAS) protein expression (% of control) in the rat liver following acute administration of olanzapine (1 mg/kg), clozapine (12 mg/kg) or vehicle (control). Data presented as mean ± SEM, (*n* = 4–6/group). *p < 0.05 vs controls

To investigate potential mechanisms underlying the altered sphingolipid concentrations in the liver we examined protein expression levels of CerS2 and ELOVL1. There was a significant effect of treatment on the level of CerS2 protein expression (F_2,14_ = 6.94, *p* < 0.01), with higher expression in the clozapine group compared to the controls (*p* < 0.05) and no change in the olanzapine group (Fig. 4). There was a trend toward a significant treatment effect in the levels of ELOVL1 expression (F_2,9_ = 3.36, *p =* 0.08), with a + 37% increase in the clozapine group compared to the controls (Fig. 4). In addition, there were no significant differences in the level of total and phosphorylated ACC1 and FAS between the antipsychotic treatment groups and the controls (all *p* > 0.05, Fig. 4).

#### ER stress markers

As ceramide synthesis occurs in the ER [47] and ER stress has been associated with hyperglycemia [14], we examined phosphorylation of IRE1 and eIF2α, two of the main initiation factors of the unfolded protein response. There were no significant treatment effects on IRE1 and Elf2a phosphorylation (*p* > 0.05), although phosphorylation of eIF2α was 52.5% higher in the clozapine group compared to the controls (Fig. 4).

## Discussion

The pathogenesis of insulin resistance and DM is highly complex and this complexity is compounded when diabetes occurs as a side-effect of antipsychotic drug treatment. This study aimed to capture changes that occur within the first 1-h post-antipsychotic drug administration in an effort to understand the initial drug impacts that could lead to the progression of metabolic side-effects during chronic treatment. This study provides the first evidence that acute clozapine administration significantly disrupts sphingolipid levels in the liver, with a robust reduction in hepatic ceramide and sphingomyelin content, and lipid species-specific effects, without any respective changes in skeletal muscle. Interestingly, the substantial clozapine-induced decrease in hepatic sphingolipids was accompanied by increased CerS2 and Elovl1 protein content, suggesting a potential compensatory reaction of the liver in an attempt to counter-regulate the drastic non-physiological decrease in ceramide and sphingomyelin content.

This study coincides with the findings of McClay et al., [30] who observed significantly reduced levels of sphingolipid metabolites, specifically sphinganine, sphingosine and phospocholine, in brain homogenates of male mice administered haloperidol (a ‘typical’ first generation antipsychotic drug) for 28-days, suggesting an overall decrease in sphingolipid content in the brain following haloperidol administration. We provide the first evidence that antipsychotic drugs interfere with ceramide and sphingomyelin levels in the liver, even after an acute dose; however, the specific effects of acute antipsychotic administration on the brain require further investigation. Given the high sphingomyelin content in the myelin sheathing that surrounds axons, McClay et al., [30] concluded that the haloperidol-induced depletion of sphingolipids was indicative of demyelination in the brain. Narayan et al., [21] investigated post-mortem prefrontal cortex samples from schizophrenia subjects who were treated with typical antipsychotic drugs and grouped based on time from initial diagnosis (i.e. recent diagnosis: < 5 years, or chronic illness: > 5 years). They reported alterations in 18 genes that encode proteins of the sphingolipid metabolism pathway in the recent diagnosis cohort but not the chronic disease cohort, with further real-time PCR analysis identifying reduced expression of enzymes that produce ceramide from both the de novo and salvage synthesis pathways compared to the controls [21]. The authors acknowledged that the inability to exclude the effects of the typical antipsychotic drugs was a limitation of the study, but suggested that the lack of changes in the chronic schizophrenia cohort may indicate that antipsychotic drugs had reversed the sphingolipid dysfunction [21]. Our results suggest that clozapine might have an acute effect on the healthy brain; however further studies examining chronic treatment effects on the brain in healthy compared to schizophrenia cohorts would provide further insight. Interestingly, antipsychotic naïve first episode schizophrenia patients also exhibit altered peripheral (skin) ceramide profiles, with a lower total ceramide fraction, but an increase in the concentration of particular ceramide species in the schizophrenia patient cohort compared to the controls [48]. In addition, analysis of prefrontal cortical and hippocampal samples from *Df(16)A+/−* mice, a model of 22q11.2 chromosome deletion (a major genetic risk factor of schizophrenia), identified alterations in ceramide phosphoethanolamines and sphingomyelin levels [49]. Overall, our findings combined with the literature demonstrate alterations in central and peripheral sphingolipids in first episode and recently diagnosed schizophrenia, preclinical schizophrenia modelling, and as a result of acute antipsychotic drug treatment (also see [29]).

In the present study, we examined the liver and skeletal muscle due to their role in metabolism and glucose homeostasis. Clozapine decreased ceramide and sphingomyelin levels in the liver, while olanzapine increased hepatic sphingomyelin and neither drugs altered lipids in the muscle. Further investigation is required in order to understand why clozapine, and to a lesser extent olanzapine, targeted sphingolipid metabolism in the liver and not the skeletal muscle. It is possible that effects were observed after 1-h in the liver as both antipsychotics are primarily metabolised by cytochrome P450 enzymes in the liver [50]. These results could also suggest that clozapine acutely increases hepatic glucose output related to altered sphingolipid metabolism in the liver, whereas sphingolipid changes in the muscle could be related more closely to peripheral insulin resistance associated with chronic treatment conditions. Indeed, hyperglycemia and significantly lowered hepatic glycogen content was evident in the acute clozapine treatment group compared to the controls, suggesting an effect of clozapine on hepatic glucose output. Increased hepatic glucose output induced by clozapine is consistent with previous reports [8–10]. The lack of hyperglycemia in the olanzapine group was contrary to our previous findings that olanzapine increased fasting glucose levels after 14-days of treatment [39, 51], and other studies of acute treatment in rats [8], likely due to differences in treatment period and dose (7.5 mg and 15 mg olanzapine in Boyda et al., [8]). Nevertheless, it enabled examination of hepatic sphingolipid levels following acute administration of two antipsychotic drugs with similar characteristics, in the absence and presence of hyperglycemia. Olanzapine is a thienobenzodiazepine derivative with structural and pharmacodynamic similarities to clozapine [52]. They have similar receptor binding profiles in the brain and both have the highest liability for causing metabolic side-effects [2, 52, 53]. However, there are clinical distinctions between these two antipsychotics as clozapine can be an effective therapy for treatment resistant schizophrenia [54], suggesting some difference in mechanism of action compared to olanzapine. An important finding of the present study is that the drugs cause different effects during the acute treatment phase. The lack of hyperglycemia in the olanzapine group could be related to the general lack in changes in hepatic sphingolipid metabolism in this group compared to the clozapine group. Future studies that examine treatment effects over time may be beneficial in order to determine whether olanzapine and clozapine have the same effects on the parameters examined in the present study after a longer period of time. On the other hand, numerous reports examining the mechanisms and prevention of antipsychotic-induced metabolic side-effects have focussed on long-term treatment effects [2, 39, 51, 55–60], but this study revealed that antipsychotics can cause alterations to metabolic parameters even after 1 h. By identifying the initial disruptions caused by olanzapine and clozapine in the acute treatment phase, we can enhance understanding of the mechanisms underlying metabolic side-effects that manifest during long term treatment.

In order to understand a potential mechanism by which clozapine and olanzapine alter longer chain sphingolipid levels, we examined protein expression of CerS2 and ELOVL1. CerS2 yields C22–24 ceramide species [22] and regulates ELOVL1 that synthesises long (C18-C26) fatty acid chains [25]. CerS2 and ELOVL1 knockdown reduces endogenous C24 sphingolipid levels in-vitro [25, 47]. Indeed, we observed reduced ceramide and sphingomyelin 24:0, 24:1 and 24:2 levels in the clozapine treatment group, but a paradoxical increase in both CerS2 and ELOVL1 protein expression levels, which may be a compensatory mechanism attempting to restore lipid homeostasis. The dramatic reduction in ceramide levels by clozapine, an obesogenic/diabetogenic antipsychotic drug, contrasts with the evidence that increased ceramide levels are a cause of diet-induced non-alcoholic fatty liver disease, while reduced hepatic ceramide levels increases insulin sensitivity [18, 19, 27, 61, 62]. In addition, one study showed that hepatic CerS2 haploinsufficiency increased susceptibility of mice to diet-induced hepatic steatosis [63], while clozapine increased hepatic CerS2 protein expression in the present study. These differences may be reconciled by the fact that the present study utilised an acute treatment paradigm. It is possible that the dramatic reduction in ceramide caused by clozapine is an initial disruption to sphingolipid homeostasis that results in a compensatory (inappropriate) upregulation of ceramide synthesis during chronic treatment. The upregulated CerS2 and ELOVL1 enzyme expression supports this notion; however, further investigation is required. On the other hand, olanzapine increased sphingomyelin levels, which could be an early indicator of hepatic lipid accumulation in line with previous reports that olanzapine promotes lipid accumulation in adipocytes and the liver [5, 7, 13, 39].

The present study has revealed early disruption of sphingolipid metabolism preceding antipsychotic-induced body weight gain. One possible explanation for the reduction in ceramide and sphingomyelin species in the clozapine group is their conversion to other lipid metabolites. For example, ceramide can be converted to sphingosine or glucosylceramide. As mentioned, sphingosine is reduced in haloperidol-treated mouse brains [30] whereas glucosylceramide levels are upregulated in the retinas of hyperglycaemic diabetic rats [64], but enzymes catalysing the conversion of ceramide to glucosylceramide are unaltered in typical antipsychotic drug-treated schizophrenia brain tissue [21]. Overall, despite the complexity of the pathways surrounding ceramide metabolism [20], further investigations into the effects of antipsychotic drugs on other components and regulators of the sphingolipid metabolism pathways are justified.

Hyperglycemia can induce ER stress, which is considered a major contributor to hepatic insulin resistance by favouring de novo lipogenesis (leading to hepatic lipid accumulation), and interfering in insulin signalling, in part, through IRE1/c-Jun N-terminal kinase (JNK) pathway activation (leading to hepatic insulin resistance and DM) [14, 65]. In addition, the ER is a site for ceramide synthesis and fatty acid elongation [7, 22, 47]. Therefore, we examined whether ER stress may have impacted ceramide levels in the present study. While some evidence shows that hepatic CerS2 overexpression reduces ER stress [12] other data show that decreased complex sphingolipids increases ER stress [26]. We did not observe significant changes in IRE1 phosphorylation, but did observe a 52.5% increase in the eIF2α phosphorylation, indicating a clozapine-induced activation of the PERK-Elf2a ER stress pathway.

## Conclusions

This study provides the first evidence that antipsychotic drugs with a high risk of causing metabolic side-effects, olanzapine and clozapine, disrupt sphingolipid homeostasis in the liver within one hour of administration. Clozapine causes a robust reduction in hepatic ceramide and sphingomyelin levels, coupled with a potentially compensatory upregulation of ceramide synthesising enzymes (CerS2 and ELOVL-1), in the presence of hyperglycemia and evidence of hepatic ER stress. On the other hand, olanzapine increased hepatic sphingomyelin levels, which is consistent with its ability to increase hepatic lipid accumulation. A limitation of this study was that we examined female rodents, which confer a greater sensitivity to metabolic side-effects than males [33]. Therefore, examination in males is required. In addition, the estrus stage of the individual rats could impact glucose metabolism through effects of estrogen and progesterone [66]; however, the small standard error of the mean in the glucose and glycogen data sets of the present study suggest low within-group variability. In addition, further time-dependent studies are required to elucidate the causal mechanism underlying this antipsychotic-induced lipid dysfunction in the acute treatment period in order to prevent the possible consequences, such as hepatic insulin resistance, lipid accumulation and DM, observed in treated individuals during chronic antipsychotic treatment.
